# Supplementary material for: CCL3 Promotes Germinal Center B Cells Sampling by Follicular Regulatory T Cells in Murine Lymph Nodes
Source: Front Immunol. 2018 Sep 13;9:2044. doi: 10.3389/fimmu.2018.02044 (PMC6146081; doi:10.3389/fimmu.2018.02044)
Supplement: Supplementary file 5 [file Data_Sheet_1.PDF]

# SUPPLEMENTARY FIGURES:

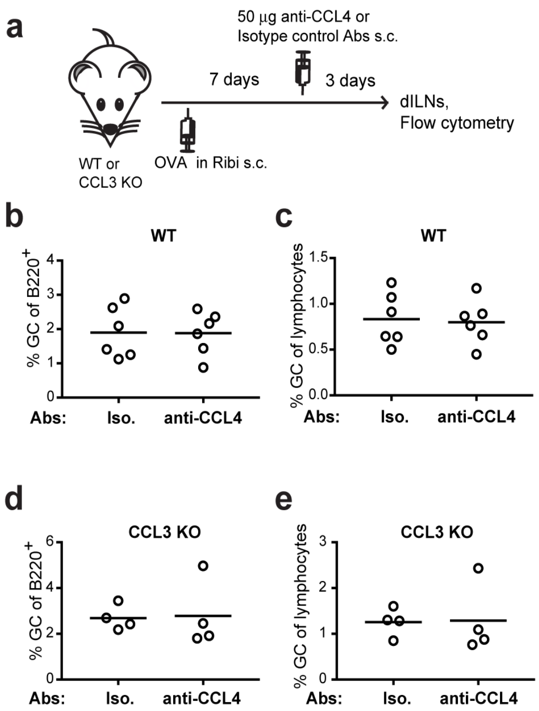


**Supplementary Figure 1. CCL4 neutralization in GCs does not amplify GC response in WT or CCL3-KO mice. a**, Experimental strategy. **b-e**, GC B cells ($B220^{pos}$ $CD4^{neg}$ $CD8^{neg}$ $FAS^{high}$ $GL7^{high}$) in the dLNs of WT (b, c) and CCL3-KO (d, e) mice as a fraction of $B220^{+}$ cells (b, d) or lymphocytes (c, d) at 10 d.p.i. with OVA in Ribi.

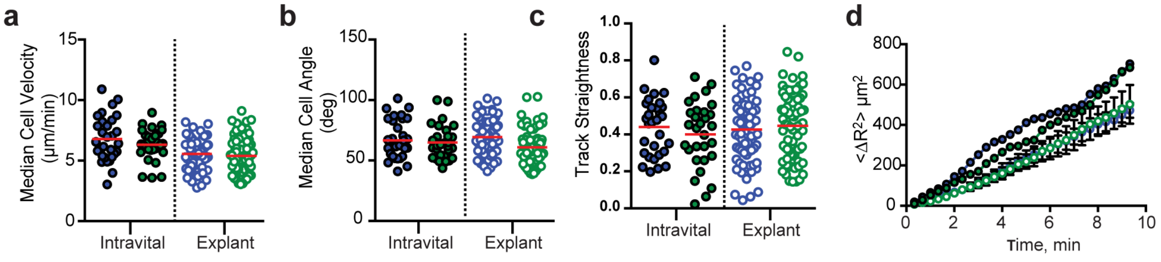


**Supplementary Figure 2: HyHEL10 GC $CCL3^{+/+}$ CFP (blue circles) and $CCL3^{-/-}$ GFP (green circles) cell motility analysis. Red lines represent medians. Data are from 4 independent experiments.**

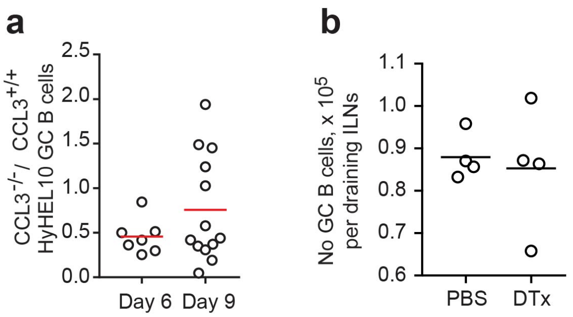


**Supplementary Figure 3: B cell participation in GCs in PBS or DTx-treated immunized mice.**
**a**, The ratio of $CCL3^{-/-}$ to $CCL3^{+/+}$ HyHEL10 GC B cells ($CD19^{+}CD8^{-}CD4^{-}$ $Fas^{high}$ $GL7^{high}$ $IgD^{low}$) at 6 and 9 d.p.i. in dILNs of $FoxP3^{DTR}$ recipient mice treated for 3 days with PBS. **b**, The number of GC B cells ($B220^{+}CD8^{-}CD4^{-}$ $Fas^{high}$ $GL7^{high}$ $CD38^{low}$ $IgD^{low}$) at 9 d.p.i. in dILNs of B6 CD45.1 mice treated with DTX or PBS for 3 days as in Fig.8. The data is from 5 (in a) and 3 (in b) independent experiments. Each dot represents a single mouse and lines correspond to the mean values.

## SUPPLEMENTARY MOVIE LEGENDS

**Movie S1. 2-photon imaging of Tfr cells migration in respect to Tfh cells and GC B cells.** Also see **Figure 5e**. A time-lapse sequence of a 110 µm z-stack of a GC in an explanted inguinal lymph node imaged at 8 days after immunization. HyHEL10 GC B cell; cyan. Tfr; red. Tfh; green. Colored lines indicate the trajectories of the indicated cell types, tracked by Imaris and manually verified. Mice were generated as described in the **Figure 5a**. Time is shown as hh:mm:ss and z-stacks were acquired at 20 second intervals.

**Movie S2. Intravital imaging of Tfr cells in respect to the GCs containing both CCL3$^{+/+}$ and CCL3$^{-/-}$ HyHEL10 B cells.** The data shown corresponds with the images in **Figure 6**. A time-lapse sequence of a GC within inguinal LN of a mouse prepared as described in **Figure 6a** and subjected to intravital imaging at 8 days post immunization. 180 µm z-stack. GC volume (gray surface) was defined based on the distribution of CFP HyHEL10 cells. Quantitative analysis in **Figure 6e-l** was performed for Tfr cells (red) interactions with HyHEL10 GC B cells (CCL3$^{+/+}$; cyan. CCL3$^{-/-}$; green.) within GC volumes defined in the same fashion. The tracks of individual Tfr cells outside the GC are labeled in purple while interior tracks are represented in yellow lines. Time is expressed as hh:mm:ss and z-stacks were acquired at 20 second intervals.

**Movie S3. Examples of Tfr cells interactions with GC B cells identified as "strict" or "nonstrict" for quantitative analysis.** A time-lapse sequence of a representative Tfr (red) entering the GC (dashed white line) and then undergoing contacts with HyHEL10 CCL3$^{+/+}$ (cyan) or CCL3$^{-/-}$ (green) HyHEL10 GC B cells within inguinal LN of a mouse prepared as described in **Figure 6a** and subjected to 2P imaging at 8 days post immunization. A 40 $\mu$M slice is in view. Inlets are zoomed in and 3D rotated to visualize the contact. Time is expressed as mm:ss and z-stacks were acquired at 25 second intervals

**Movie S4. 2-photon imaging of Tfh cells interacting with GC B cells**. A time-lapse sequence of a GC within an inguinal LN of a mouse prepared as described in **Figure 6b** and subjected to explant imaging at 8 days post immunization. 140 um z-stack**.** Quantitative analysis was performed for Tfh cells (red) interactions with HyHEL10 GC B cells (CCL3$^{+/+}$, cyan; CCL3$^{-/-}$, green) within the GC and can be found in **Figure 6e-l**. Examples of long duration (>5 minutes) contacts and short duration (<5 minutes) contacts are shown in 10 um z-projections. Time is shown as hh:mm:ss for both time of the movie and for duration of the indicated contacts. Z-stacks were acquired at 20 second intervals.
